# An Adolescent Boy With Hypoxia, Microscopic Hematuria, and Hypertension

**DOI:** 10.7759/cureus.52738

**Published:** 2024-01-22

**Authors:** Melissa S Zhou, Clement D Lee, Benjamin J Lerman, Alanna Strong, Christopher LaRosa

**Affiliations:** 1 Pediatrics, Children's Hospital of Philadelphia, Philadelphia, USA; 2 Genetics, Children's Hospital of Philadelphia, Philadelphia, USA; 3 Nephrology, Children's Hospital of Philadelphia, Philadelphia, USA

**Keywords:** recurrent hematuria, hematuria, microscopic hematuria, alport syndrome, asymptomatic hematuria

## Abstract

A 13-year-old boy presented with hypoxia, microscopic hematuria, and elevated blood pressures. Persistent microscopic hematuria and hypertension led to investigation of glomerular and non-glomerular causes of hematuria. After reviewing his clinical course, family history, and laboratory testing, an additional test was sent, revealing the diagnosis.

## Introduction

Hematuria is not uncommon in the pediatric population, with 1% of all school-aged children exhibiting microscopic hematuria if routinely screened by urine dipstick [1]. Because the etiology of hematuria varies from benign causes (e.g., exercise, fever) to life-threatening disease (e.g., hemolytic-uremic syndrome [HUS]), a systematic approach to hematuria is warranted to ensure all entities are considered. We describe the case of a 13-year-old boy who presented to the hospital with an infection, but his persistent hematuria was of concern and ultimately led to the diagnosis of a systemic disease with significant implications.

## Case presentation

A 13-year-old boy with a past medical history of cyclic vomiting syndrome, severe obesity, developmental delay, autism spectrum disorder, and attention-deficit/hyperactivity disorder presented to a pediatric emergency department with one day of fever to 103°F and non-bloody, non-bilious emesis without associated diarrhea. Home medications included clonidine and guanfacine.

**Figure 1:**
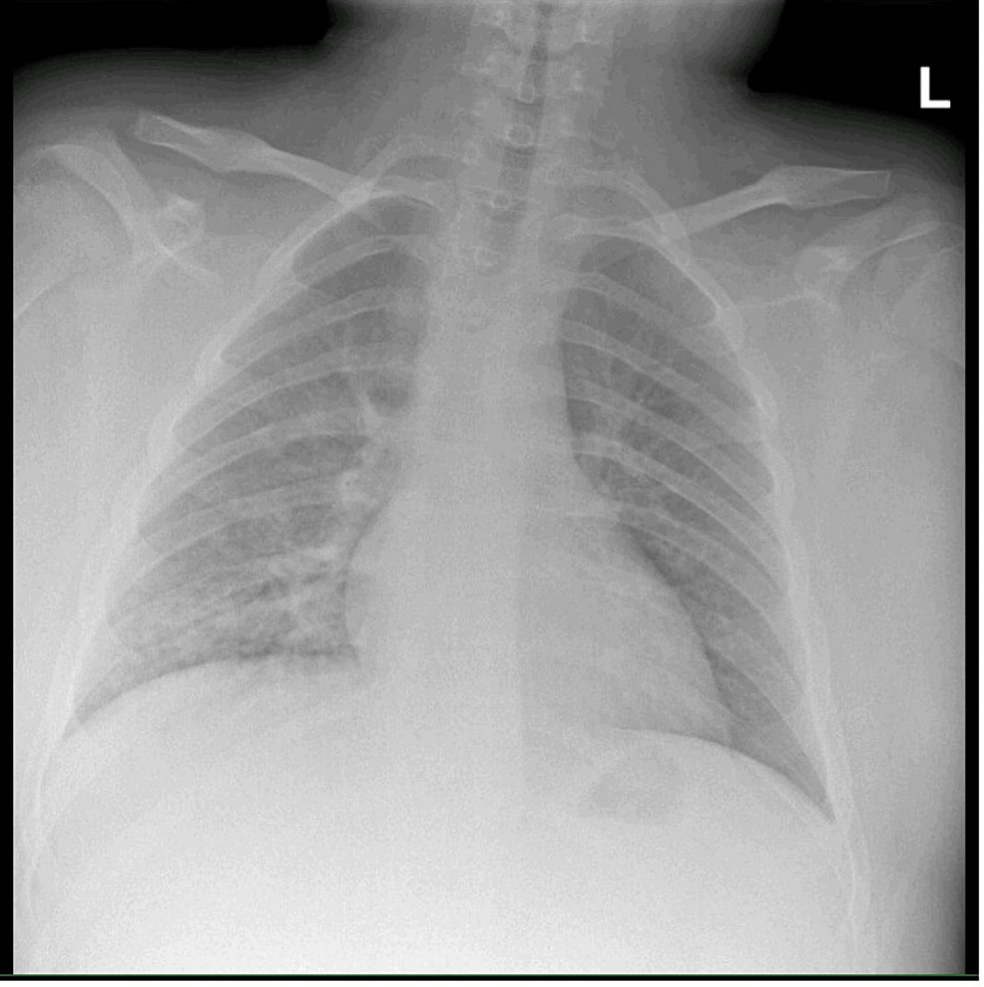
Anteroposterior chest radiograph of the patient.

**Figure 2:**
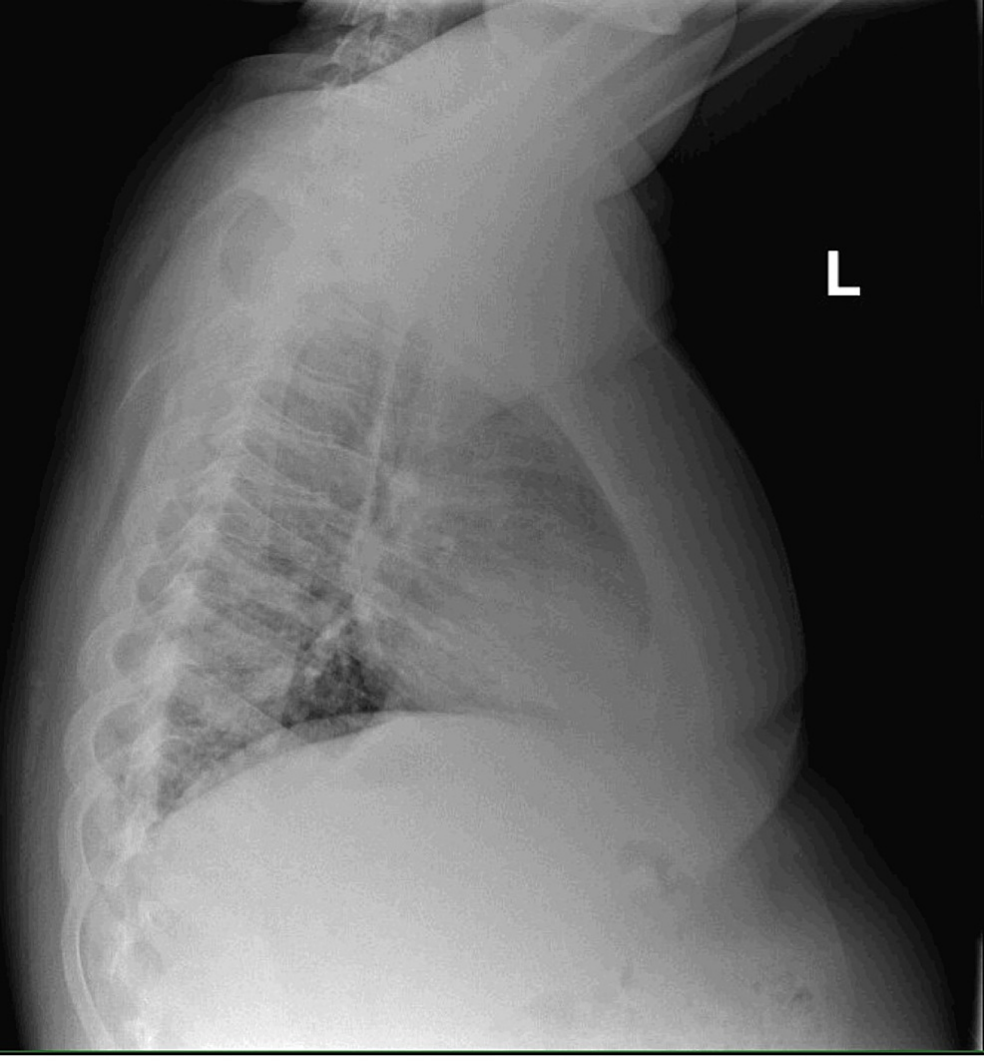
Lateral chest radiograph of the patient confirming an opacity in the right lower lobe.

On evaluation, he was afebrile, had a heart rate of 103 beats per minute, blood pressure of 148/64 mmHg, and a peripheral oxygen saturation of 88% in ambient air. He was intermittently retching and reported tenderness to palpation of his epigastrium. Laboratory examination showed a white blood cell count of 13.2 K/uL (reference range: 3.8-9.8 K/uL), creatinine of 0.7 mg/dL (reference range: 0.3-0.8 mg/dL), and normal serum electrolytes, liver enzymes, and lipase. Dipstick urinalysis from a clean-catch specimen was notable for large blood, protein >300 mg/dL, ketones, specific gravity of >1.030, and normal pH. Urine nitrates and leukocytes were negative. A chest radiograph demonstrated a right lower lobe airspace opacity consistent with pneumonia (Figures 1, 2). Nasal polymerase chain reaction (PCR) testing for influenza A and B was negative. The patient was admitted for management of community-acquired pneumonia.

During the admission, the patient continued to have intermittent elevated blood pressures, with systolic measurements reaching 170-180 mmHg. Repeat urinalysis with microscopy showed 10-25 red blood cells/high-powered field and a protein/creatinine ratio of 0.28 (reference range: <0.2). A urine calcium-to-creatinine ratio and levels of C3/C4 complements were normal. A renal bladder ultrasound with Doppler demonstrated normal appearance, size, and vascular flow of both kidneys. He was discharged with a seven-day course of amoxicillin/clavulanate for community-acquired pneumonia and referred to the outpatient nephrology clinic for persistent hematuria and hypertension.

He was readmitted two days later with worsening respiratory symptoms. Repeat workup was notable only for stable mild normocytic anemia (hemoglobin level of 11.4 g/dL; reference range: 13.0-16.0 g/dL) and a nasopharyngeal PCR that was positive for *Mycoplasma pneumoniae*. He was administered azithromycin for a presumed *M. pneumonia* that eluded coverage by the prior antibiotic prescription. He continued to have microscopic hematuria with large blood on urinalysis. Levels of anti-neutrophil cytoplasmic antibody (ANCA), markers of hemolysis, and creatinine kinase were all normal.

Outpatient nephrology evaluation two months later showed persistent microscopic hematuria and elevated blood pressure, with systolic blood pressures in the 99th percentile for age. Family history was notable for hematuria and proteinuria in the patient’s father and identical twin brother, isolated hematuria during pregnancy in the mother, and frequent nephrolithiasis in the father. With this family history and ongoing microscopic hematuria, a gene panel was sent to evaluate for causes of hereditary nephritis. Our patient was found to have a variant of uncertain significance in the *COL4A5 *gene. Subsequent whole exome sequencing confirmed this variant in the patient, his twin brother, and his mother. A diagnosis of X-linked Alport syndrome was made.

The patient was started on lisinopril to preserve kidney function and referred for both audiologic and ophthalmologic testing. On subsequent follow-up visits, the patient had normal estimated glomerular filtration rates, blood pressures, and electrolytes without evidence of albuminuria.

## Discussion

At first presentation, this patient demonstrated hematuria, proteinuria, and elevated blood pressures in the setting of fever and infection. Transient hematuria and/or proteinuria can be seen with illness. When hematuria is demonstrated for the first time, urinalysis should be repeated, as results may be confounded by trauma (e.g. catheterization), exercise, menstruation, or sexual activity. In this case, the history does not support any of these circumstances. Detection of protein on urinalysis must also be contextualized; urine that is alkaline, is concentrated, or contains a large amount of blood can be falsely positive for protein. Orthostatic proteinuria should be ruled out with a first morning void.

The constellation of hematuria, proteinuria, and elevated blood pressures should raise concern for glomerulonephritis (GN). The differential diagnosis for GN includes hypocomplementemic etiologies (e.g., post-infectious GN, membranous proliferative GN, systemic lupus erythematosus, C3 glomerulopathy) and normocomplementemic etiologies (e.g., immunoglobulin A nephropathy, antineutrophil cytoplasmic autoantibody (ANCA)-associated vasculitis, Alport syndrome). Many of these disease processes can present with intercurrent pulmonary symptoms such as those that this patient experienced. Non-glomerular etiologies of hematuria in the setting of this patient’s presentation, including *M. pneumoniae* infection-related sequalae (renal vascular embolic event, urethral mucositis associated with *M. pneumoniae*-induced rash and mucositis), pneumococcal-associated HUS, and urethral irritation from nephrolithiasis, were less likely. Alport syndrome is an important potential cause of persistent microscopic hematuria in children and adolescents, especially when there is an parental family history of hematuria. The genetic testing performed on his twin brother and mother, along with his compatible clinical history, confirms the diagnosis.

Alport syndrome is the second most common cause of monogenic kidney disease, after autosomal dominant polycystic kidney disease, and is caused by pathogenic variants in the *COL4A3*, *COL4A4*, and *COL4A5* genes encoding the alpha chains of type IV collagen in glomerular, cochlear, and ocular basement membranes. The majority of cases of Alport syndrome are caused by hemizygous/heterozygous variants in *COL4A5* and demonstrate X-linked inheritance, while remaining cases of *COL4A3* or *COL4A4*-related Alport syndrome are inherited in an autosomal dominant or recessive manner [2]. Alport syndrome is responsible for an estimated 3% of chronic kidney disease (CKD) in children and 0.2% of end-stage kidney disease (ESKD) in adults [3]. There is inter- and intrafamilial variability in presentation depending on genotype, sex (especially for X-linked Alport syndrome), and other factors. Microscopic hematuria is nearly universal and is often discovered incidentally. Routine screening urinalysis in childhood is not the standard of practice in the United States but is performed in other countries such as Japan.

Alport syndrome generally progresses through predictable phases of kidney pathology, from isolated hematuria to microalbuminuria, then to overt proteinuria, and ultimately to kidney function decline. The rate of progression to ESKD, like the development of the extra-renal manifestations, can vary by genotype and patient sex. Progression to ESKD is seen most often in those with X-linked and autosomal recessive Alport syndrome and typically occurs in the third to fourth decades of life. Renal transplant is the standard of care for ESKD in patients with Alport syndrome, and outcomes are generally favorable. The development of antibodies to type IV collagen alpha chains may occur post-transplant but is rare and seen mostly in male X-linked and autosomal recessive Alport syndrome. The clinical correlate to these antibodies is anti-glomerular basement membrane (GBM) disease and may present with GN that leads to graft loss.

The American Academy of Pediatrics revised its screening guidelines in 2007 to remove universal childhood urinalysis screening in asymptomatic children due to the low incidence of CKD in children, the lack of benefit in early identification of CKD in asymptomatic children, and overall cost-ineffectiveness [4]. However, children at high risk for CKD, such as those with a family history of genetic renal disease or signs and symptoms consistent with one, should be screened. A high index of suspicion for Alport syndrome should be maintained in children who have a family history of hematuria, hearing loss, and/or kidney disease. Early initiation of renin-angiotensin-aldosterone system inhibitor (RAASi) therapy once a diagnosis has been made is important to delay progression of kidney function decline [5].

Alport syndrome has auditory and ocular manifestations. The age of onset of hearing loss is variable depending on genotype and sex, but, in general, formal hearing screening should be started around five to six years of age and repeated every one to two years for male patients with X-linked Alport syndrome. Monitoring for ocular anomalies should also be completed every one to two years, beginning in the teen years for those with X-linked Alport syndrome. Characteristic findings include fleck retinopathy, cataracts, and anterior lenticonus. While fleck retinopathy is asymptomatic, anterior lenticonus, if present, can lead to vision impairments requiring eyeglasses [6].

## Conclusions

In our patient, timely referral for hematuria led to the diagnosis of Alport syndrome before the onset of kidney function decline. As clinicians, we must be vigilant when we “see red” and not ignore hematuria, as careful consideration of its causes and systematic approach to evaluation can lead to more expedient diagnosis and better renal outcomes for our patients.
